# Surgical induced necrotizing scleritis following intraocular lens replacement

**DOI:** 10.1186/s12348-023-00373-y

**Published:** 2023-12-07

**Authors:** Pablo González de los Mártires, Gonzalo Guerrero Pérez, Iñigo Les Bujanda, Iñaki Elejalde Guerra, Henar Heras Mulero, Esther Compains Silva

**Affiliations:** 1 Department of Ophthalmology, Hospital Universitario de Navarra, Pamplona, Navarra Spain; 2 Department of Ophthalmology, Hospital de Calahorra, Calahorra, La Rioja Spain; 3 Department of Internal Medicine, Hospital Universitario de Navarra, Pamplona, Navarra Spain

**Keywords:** Surgery-induced necrotizing scleritis, Anterior segment inflammation, Anti-TNF-α, Adalimumab, Scleral patch graft

## Abstract

**Purpose:**

To report a surgical-induced necrotizing scleritis, as well as its medical and surgical management.

**Methods:**

Case-report.

**Results:**

An 88 year-old patient with a three-day severe single-left-eye ocular pain. One-time surgery involving PPV with removal of dislocated intraocular lens and secondary implantation of iris-claw Artisan® lens was performed 6 months earlier. Visual acuity of 20/100. Slit-lamp examination revealed a 5 × 2 mm non-suppurative superior scleral defect. Empirical topical antibiotic treatment with dexamethasone, as well as oral doxycycline was started. Infectious and autoimmune diseases were ruled out. Non-infectious scleritis treatment was conducted with intravenous Methylprednisolone 3 day pulses, followed by weekly tapered Prednisone and intramuscular Methotrexate. However, 1 month after the diagnosis, the defect was worsened; hence, a heterologous scleral patch graft was performed and, days after the intervention, Adalimumab was initiated. To date, 6 months later, remains with proper scleral patch, a diary low-dose Prednisone, and spacing Adalimumab treatment.

**Conclusion:**

Surgery-induced necrotizing scleritis is a severe condition that compromise the ocular and visual integrity. Proper diagnosis, as well as early treatment is required to achieve remission, prevent relapses, and avoid structural complications. In refractory cases, anti-TNF-α immunotherapy associated with surgical tectonic graft interventions can achieve promising results.

**Supplementary Information:**

The online version contains supplementary material available at 10.1186/s12348-023-00373-y.

## Introduction

Surgery-induced necrotizing scleritis (SINS) is a rare local autoimmune reaction of the sclera adjacent to previous surgical incisions, which has been reported following cataract surgery, trabeculectomy, scleral buckling, as well as pterygium and strabismus surgery [1–4]. Ocular trauma serves as more than just a pathogenic mechanism; it acts as a triggering factor that can initiate scleral necrosis through various pathogenic pathways. Patients who develop SINS exhibit an atypical response to surgical trauma, which can be attributed to the presence of unusual local and systemic immunological factors [1].

We present the case of a patient with SINS following vitrectomy after explantation of a luxated intraocular lens (IOL) and secondary implantation of an anterior iris-fixated Worst claw Artisan® IOL. A systemic workup was conducted to rule out underlying pathologies. We describe the multidisciplinary medical management as well as the surgical approach. Slit lamp and intraoperative images are provided to illustrate the clinical case.

## Case description

An 88-year-old woman with a history of insulin-dependent diabetes mellitus, stage 3a chronic kidney disease (CKD), bilateral atrophic age-related macular degeneration (ARMD) and advanced glaucoma in her right eye, along with four months of nonspecific joint pain without clear signs of inflammation, sought medical attention.

She exhibited a spontaneous posterior dislocation of her left eye intraocular lens (IOL) and subsequently underwent a pars plana vitrectomy. During the procedure, the dislocated IOL was removed, and a secondary implantation of a prepupillary iris-claw Artisan® IOL was performed through a 5.5 mm superior sclerocorneal incision. The surgery was successfully completed, and the immediate postoperative recovery period was free from any complications. Several weeks later, the four Nylon 10/0 sutures used during the surgery were removed.

**Fig. 1:**
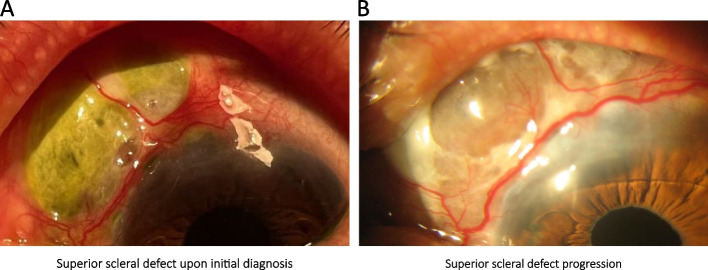
**A** Superior scleral defect upon initial diagnosis. **B** Superior scleral defect progression

Nonetheless, six months following the medical intervention, she presented at the emergency room with acute ocular pain in her left eye that had persisted for three days. Her visual acuity had deteriorated to 20/100 (previous 20/40). A slit-lamp examination revealed a non-suppurative defect in the superior sclera measuring 5 × 2 mm (see Fig. 1A). Her intraocular pressure was within the normal range, and the fundus examination showed no other abnormalities. Based on the clinical findings, a diagnosis of anterior necrotizing scleritis was made, with uncertainty about whether it was due to infection or related to the prior surgery. Empirical treatment was initiated, including Tobramycin-Dexamethasone eye drops every 8 hours, Moxifloxacin 0.5% eye drops every 4 hours, Atropine 1% eye drops twice a day, and oral Doxycycline 100 mg every 12 hours.

The patient was then referred to the multidisciplinary ocular inflammation clinic at the University Hospital of Navarra, where a comprehensive systemic evaluation was performed. This evaluation included a complete blood count with biochemical analysis, C-reactive protein, rheumatoid factor, complement fractions, and serological tests, as well as testing for specific antigens (HLA-B27, HLA-B51, HLA-A29) and autoantibodies (such as antinuclear, antineutrophil cytoplasmic, or anti-citrullinated antibodies, among others). A tuberculin test and chest X-ray were also conducted. However, these tests did not reveal any underlying infectious or autoimmune systemic disorders. Additional tests, such as PCR analysis of the aqueous humor sample and conjunctival scrape for microbiological culture, were negative for bacteria, fungi, and atypical mycobacteria.

Consequently, a definitive diagnosis of surgery-induced anterior necrotizing scleritis was established, leading to the discontinuation of the empirical treatment, except for oral Doxycycline. The patient was then started on intravenous systemic treatment with Methylprednisolone succinate at a daily dose of 60 mg for three days (1 mg/kg/day), followed by a weekly tapering dose of oral Prednisone at 30 mg/day. Methotrexate subcutaneous injection at a dose of 7.5 mg per week was also initiated.

Unfortunately, the patient experienced a significant increase in blood sugar levels, prompting a reduction of Prednisone to 10 mg per day and an increase in Methotrexate to 12.5 mg per week. This exacerbated the ongoing scleritis, necessitating the resumption of Methylprednisolone in three-day boluses, this time at a dose of 90 mg (1.5 mg/kg/day), with strict monitoring and control of blood sugar levels.

**Fig. 2:**
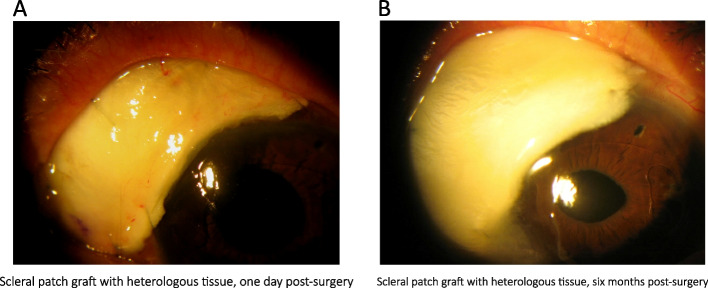
**A** Scleral patch graft with heterologous tissue, one day post-surgery. **B** Scleral patch graft with heterologous tissue, six months post-surgery

Despite these interventions, one month after the initial diagnosis, and due to the progressive nature of the scleral defect (see Fig. 1B), a heterologous scleral patch graft surgery was performed following meticulous debridement of the adjacent necrotic tissue (see Fig. 2A & Video). Additionally, a second esclerocorneal scrape was performed, but the microbiological results remained inconclusive.

To manage postoperative inflammation, subcutaneous Adalimumab was introduced after the surgery, starting with an 80 mg dose, and gradually reducing it to 40 mg in subsequent weeks. Methotrexate was discontinued from the second week post-surgery, and a new tapering regimen of Prednisone at 30 mg per day was initiated, this time with careful spacing. Six months after the surgery, the scleral patch remained stable (see Fig. 2B), and the patient continued with Prednisone at 7.5 mg/day and Adalimumab at a dosage of 40 mg every six weeks.

## Discussion

Surgery-induced necrotizing scleritis (SINS) can be triggered by any type of ocular surgical intervention, and the latency period is highly variable, ranging from first postoperative day to years after surgery [1]. Women are more affected, and the average age is fifth decade of life [1]. Pterygium excision is the most associated ocular surgery and autoimmune diseases are specially related in the cataract extraction cases [1, 2].

Predicting SINS poses a considerable challenge, as postoperative scleral thinning can go unnoticed, leading to a delayed diagnosis. In the early stages of the condition, patients may encounter symptoms like conjunctival redness and mild discomfort, which are frequently misinterpreted as dry eye disease or conjunctivitis, prolonging the diagnosis and treatment process [1]. In advanced cases, patients typically manifest significant pain, visual impairment, peripheral corneal ulcers, scleral necrosis, and the development of anterior staphylomas [1].

This entity involves a common induction mechanism (ocular surgery) and a characteristic pathological finding (scleral necrosis) [1]. Physical trauma caused by excessive cauterization or cryotherapy, chemically induced damage with antimetabolites, compression-related ischemia, like tighten sutures, as well as autoimmune local activation, represent a wide range of pathogenic mechanisms that can induce scleral necrosis [1, 2, 5]. Diabetes is also related due to the pro-ischemic inflammatory environment that exerts [6]. All these induce a slowdown in surgical wound healing, that can lead to a secondary infection [7]. The overlap between these mechanisms complicates the diagnosis and its corresponding treatment.

Considering the multiple aetiologies related to necrotizing scleritis, a comprehensive study should be conducted to discard primary infectious scleritis or underlying autoimmune involvement [5]. The research should include complete blood count, erythrocyte sedimentation rate, C-reactive protein, liver and kidney function tests, serum complement levels, antinuclear antibody, rheumatoid factor, antineutrophil cytoplasmic antibody, anti-citrullinated protein antibody, and serum angiotensin-converting enzyme levels [5]. To rule out infectious involvement, wound scrape for microbiological culture, or for scleral biopsy, and intraocular samples for PCR are necessary [2, 5, 7]. In addition, testing for tuberculosis, syphilis, HIV infection, hepatitis B and C, herpes simplex and varicella-zoster viruses should be performed [2, 7].

Due to the devastating consequences for the eye, early detection and treatment are fundamental. Topical antibiotics and/or antifungals are often empirically administered [1, 2]. The presence of mucopurulent discharge or hypopyon increases suspicion of an infectious aetiology, thus initiating topical and systemic antibiotic treatment is recommended [7]. Topical corticosteroids are associated with poor wound healing [1, 8]. Therefore, they have the potential to perpetuate the scleral thinning process, as it happens in the early stages of our case. As adjunctive treatment, oral doxycycline (100 mg/day) can also be added for its anticolagenolytic effect [8].

Systemic nonsteroidal anti-inflammatory drugs are useful at diagnosis [1, 5]. However, long-term use is not recommended due to their side effects [8]. Oral prednisone is considered first-line therapy in non-infectious necrotizing scleritis, with an initial immunosuppressive dose of 1 mg/kg/day gradually tapered based on clinical recovery [1, 5, 8]. Due to the stage 3a CKD of our patient, the corticosteroid doses were inferior to usual. Intravenous methylprednisolone for 3 days has shown efficacy in patients with severe inflammation, as it happens in our case [1, 8]. A patient with SINS will require a combination with nonsteroidal immunomodulatory therapy to enhance efficacy and avoid the long-term adverse effects of corticosteroids [1, 5, 8]. Some medications used include methotrexate, azathioprine, mycophenolate mofetil, cyclophosphamide cyclosporine and tacrolimus [5, 8, 9]. We choose the first one due to his safety profile in elderly and comorbidly patients [8].

A better understanding of the immunopathogenesis of ocular inflammatory diseases, as well as the development of new molecules, has enriched our therapeutic arsenal and created more effective treatment approaches [10, 11]. Monoclonal antibodies against TNF-α (all except Etanercept), IL-1 inhibitors (Anakinra), IL-6 inhibitors (Tocilizumab), and anti-CD20 (Rituximab) targeted-drugs have been shown to control inflammation and reduce scleritis flares, allowing for a corticosteroid-sparing effect [5, 8, 10, 11]. TNF-α inhibitors like Adalimumab or Infliximab are used as first-line biological agents [12, 13]. Adalimumab is the only biologic treatment approved for non-infectious uveitis and it has been used in other case reports with excellent clinical outcomes [13].

However, patients with active and progressive scleral necrosis will require immediate conjunctival debridement [14]. Apart from obtaining a scrape when infection is suspected, conjunctival resection results in a decrease of protease concentration, including collagenase, and a temporary interruption of the local inflammatory process [1, 14]. The use of cyanoacrylate glue after surgical debridement can help prevent neutrophil migration [1]. Conjunctival and Tenon’s flap grafts, as well as amniotic membrane transplantation, can also be used [1]. However, when uveal tissue is exposed, a tectonic procedure with scleral patch graft is preferred [1, 5, 15].

## Conclusion

Surgery-induced necrotizing scleritis is a severe ophthalmological condition that can compromise the ocular and visual integrity. It is associated with autoimmune diseases in around 50% of cases and can be their initial manifestation, which is why a comprehensive analytical screening is necessary to rule out this possibility. Concurrent infectious involvement should also be ruled out as it influences the therapeutic management of the condition. Aggressive and early treatment is required to achieve rapid remission, prevent relapses, and ultimately avoid irreversible structural complications. Systemic corticosteroids combined with alkylating immunomodulators are positioned as first-line therapy, with anti-TNF biological drugs as the alternative option. The management of scleritis should be carried out in a multidisciplinary setting to maximize treatment benefits and minimize safety concerns. Surgical intervention is reserved for extensive defects with significant uveal exposure, despite adequate medical treatment. Amniotic membrane transplantation and heterologous scleral patch graft are the main options to consider, amongst others.

### Supplementary Information

**Additional file 1.**

## Data Availability

The data used in this case report is available from the corresponding author on reasonable request.

## Abbreviations

PPV: Pars plana vitrectomy
IOL: Intraocular lens
SINS: Surgical-induced necrotizing scleritis
CKD: Chronic kidney disease
ARMD: Age-related macular disease
HLA: Human leukocyte antigens
HIV: Human immunodeficiency virus
PCR: Polimerase chain reaction
IL: interleukin
TNF: Tumour necrosis factor
PGDLM: Pablo González de los Mártires
GGP: Gonzalo Guerrero Pérez
ILB: Iñigo Les Bujanda
IEG: Iñaki Elejalde Guerra
HEM: Henar Heras Mulero
ECS: Esther Compains Silva
